# Supplementary material for: Source Apportionment of Fine Organic Particulate Matter (PM2.5) in Central Addis Ababa, Ethiopia
Source: Int J Environ Res Public Health. 2021 Nov 4;18(21):11608. doi: 10.3390/ijerph182111608 (PMC8583055; doi:10.3390/ijerph182111608)
Supplement: Supplementary file 1 [file ijerph-18-11608-s001.zip › ijerph-1355799-supplementary.pdf]

## Supplementary Figures and Tables

### Supplemental Figures

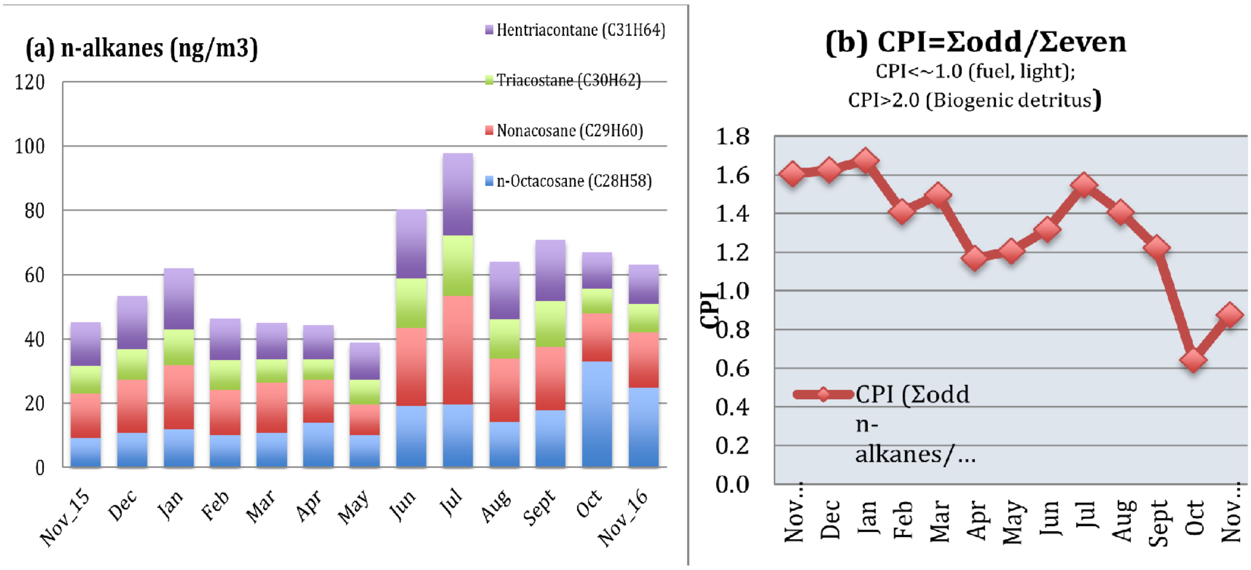


**Figure S1.** n-alkanes (a) Stacked graph of monthly n-alkanes (ngm$^{-3}$), (b) Carbon Preference Index (CPI) (Sum of Odd/Even n-alkanes).

**Table S1.** Summary of previous $PM_{2.5}$ and $PM_{10}$ source apportionment results carried out on cities in Africa.

| Study site; Study year; Site typology; Mean $PM_{2.5}$, Mean $PM_{10}$ | Population estimate (mill) | Particle size Fraction | Method-ology; sampling season | SEA SALT % | DUST % | TRAFFIC/ Motor Vehicle*** % | INDUSTRIES % | BIOMASS BURNING/ RESIDENTIAL% | OTHER (unspecified Human origin)% | Reference * |
|---|---|---|---|---|---|---|---|---|---|---|
| Accra, Ghana; 2013; urban; 66 | ~1.6 | $PM_{2.5}$ | PMF; year | | 17.0 | 22.5 | | 50.5 | 10.0 | Zhou Z; Zhou L; 2013* |
| Addis Ababa, Ethiopia; 2008; 80 | 2.8 | $PM_{10}$ | Reconstructed mass; Dry & Wet months | | 76-95 | | | | 5-24 | Gebre**, urban/suburb; 2010 |
| **Addis Ababa, Ethiopia; 2015/2016; urban; 54** | ~4.0 | **$PM_{2.5}$** | **CMB; year** | | **17.4** | **28.0*** ** | | **18.3** | **36.3** | **This study** |
| Cairo, Egypt; 2010/2011; urban; 51 | 9.12 | $PM_{2.5}$ | PCA; year | | 55.7 | 8.9 | 18.0 | 5.7 | 11.7 | Boman, 2012* |
| Cairo, Egypt; 1999/2002; urban; 86 | 9.12 | $PM_{2.5}$ | CMB; year | 2.0 | 7.0 | 22.0 | 13.0 | 30.0 | 26.0 | ESMAP Report, 2011* |
| Cairo, Egypt; 1999/2002; urban; 187 | 9.12 | $PM_{10}$ | CMB; year | 2.0 | 33.0 | 9.0 | 11.0 | 28.0 | 17.0 | ESMAP Report, 2011* |
| Dar es Salam, Tanzania; 2005/2007; rural; 13 | 1.361 | $PM_{10}$ | PCA; year | 33.8 | 17.2 | 25.8 | | | 23.2 | Mmari, 2013* |
| Dar es Salam, Tanzania; 2005/2007; urban; 14 | 1.361 | $PM_{10}$ | PCA; year | | | 63.6 | | | 36.4 | Mmari, 2013* |
| Dar es Salam, Tanzania; 2005/2007; urban; 17 | 1.361 | $PM_{10}$ | PCA; year | 29.3 | | 37.1 | 7.1 | 13.6 | 12.9 | Mmari, 2013* |
| Ikeja, Nigeria; 1989/1991; industrial; 176 | 0.313 | $PM_{10}$ | CMB; year | 27.8 | | 33.6 | 7.4 | | 31.2 | Oluyemi, 2001* |
| Ikoyi, Nigeria; 1989/1991; urban; 92 | 5.195 | $PM_{10}$ | CMB; year | 24.4 | | 57.6 | 4.5 | | 13.5 | Oluyemi, 2001* |
| Qalabotjha, South Africa; 1997; urban; 124 | ~0.016 | $PM_{10}$ | CMB; winter | | 1.0 | | 1.0 | 75.0 | 23.0 | ESMAP Report, 2011* |
| Qalabotjha, South Africa; 1997; urban; 113 | ~0.016 | $PM_{2.5}$ | CMB; winter | | 11.0 | | 2.0 | 63.0 | 24.0 | ESMAP Report, 2011* |
| Yaba, Nigeria; 1989/1991; residential; 188 | 5.195 | $PM_{10}$ | CMB; year | 34.2 | | 39.0 | 7.8 | | 19.0 | Oluyemi, 2001* |

* Source: WHO Ambient Air Quality Database, 2018; ** Gebre, G. et al., 2010; *** motor vehicles. CMB: Chemical Mass Balance; PCA: Principal Component Analysis; ESMAP: Energy Sector Management Assistance Program (World Bank).

**Table S2.** a) Organic Carbon (OC) source contribution estimates by concentration (µg OC/m³) in Addis Ababa, November 2015 through November 2016.

| Month | Measured OC | Measured OC unc | R-square | CHI-square | Biomass burning | Biomass burning unc | Vehicles | Vehicles unc | Residential coal combustion | Residential coal combustion unc | Fuel oil | Fuel oil unc | Other OC | Other OC unc |
|---|---|---|---|---|---|---|---|---|---|---|---|---|---|---|
| Nov 15 | 16.3 | 2.5 | 0.99 | 0.56 | 4.6 | 0.9 | 3.8 | 0.4 | 0.80 | 0.18 | 0.010 | 0.002 | 7.1 | 2.7 |
| Dec | 17.0 | 3.1 | 0.99 | 0.62 | 5.4 | 1.1 | 4.0 | 0.8 | 0.70 | 0.21 | 0.011 | 0.003 | *6.9* | *3.4* |
| Jan | 14.4 | 4.6 | 0.99 | 0.36 | 6.7 | 1.4 | 3.8 | 0.6 | 0.89 | 0.26 | 0.008 | 0.001 | *3.0* | *4.8* |
| Feb | 19.1 | 4.4 | 0.99 | 0.58 | 4.2 | 0.9 | 4.8 | 1.2 | 0.79 | 0.19 | 0.016 | 0.002 | 9.4 | 4.6 |
| Mar | 15.5 | 2.1 | 0.98 | 1.57 | 4.6 | 0.9 | 4.2 | 0.6 | 0.75 | 0.18 | 0.011 | 0.003 | 5.9 | 2.4 |
| Apr | 13.7 | 3.3 | 0.98 | 1.39 | 4.5 | 0.9 | 3.7 | 0.5 | 0.66 | 0.17 | 0.009 | 0.002 | *4.9* | *3.5* |
| May | 11.1 | 0.9 | 0.99 | 1.22 | 5.4 | 1.1 | 4.8 | 0.7 | 0.53 | 0.16 | 0.009 | 0.002 | *0.3* | *1.6* |
| Jun | 24.1 | 1.4 | 0.99 | 0.56 | 14.2 | 2.9 | 10.5 | 1.0 | 0.97 | 0.28 | 0.020 | 0.002 | *-1.6* | *3.3* |
| Jul | 25.9 | 5.0 | 0.99 | 0.89 | 16.1 | 3.2 | 9.8 | 1.0 | 1.61 | 0.34 | 0.013 | 0.003 | *-1.7* | *6.0* |
| Aug | 19.0 | 3.9 | 0.98 | 1.55 | 10.1 | 2.0 | 9.0 | 1.5 | 0.68 | 0.18 | 0.009 | 0.002 | *-0.8* | *4.6* |
| Sep | 22.3 | 4.2 | 0.99 | 1.09 | 11.3 | 2.3 | 9.7 | 1.3 | 0.89 | 0.20 | 0.013 | 0.002 | *0.4* | *4.9* |
| Oct | 10.2 | 1.2 | 0.99 | 0.61 | 5.6 | 1.1 | 5.7 | 0.7 | 0.28 | 0.11 | 0.012 | 0.002 | *-1.4* | *1.8* |
| Nov 16 | 11.9 | 1.6 | 0.99 | 0.49 | 8.8 | 1.8 | 6.5 | 1.0 | 0.83 | 0.19 | 0.010 | 0.002 | *-4.2* | *2.5* |

Units: micrograms of **organic carbon** per cubic meter (µg OC/m³)

*Blue italic text indicates **non-significant** values (criteria: concentration is **less than** 2*uncertainty)*

**Table S2.** b) Organic Carbon (OC) source contribution estimates by percentage (%) in Addis Ababa, November 2015 through November 2016.

| Month | Biomass burning | Biomass burning unc | Vehicles | Vehicles unc | Residential coal combustion | Residential coal combustion unc | Fuel oil | Fuel oil unc | Other OC | Other OC unc |
|---|---|---|---|---|---|---|---|---|---|---|
| Nov 15 | 28% | 7% | 23% | 4% | 4.9% | 1.4% | 0.06% | 0.02% | 44% | 18% |
| Dec | 32% | 9% | 23% | 6% | 4.1% | 1.4% | 0.07% | 0.02% | | |
| Jan | 47% | 18% | 27% | 9% | 6.2% | 2.6% | 0.05% | 0.02% | | |
| Feb | 22% | 7% | 25% | 9% | 4.1% | 1.4% | 0.08% | 0.02% | 49% | 27% |
| Mar | 30% | 7% | 27% | 5% | 4.9% | 1.3% | 0.07% | 0.02% | 38% | 16% |
| Apr | 32% | 10% | 27% | 8% | 4.8% | 1.7% | 0.06% | 0.02% | | |
| May | 49% | 11% | 44% | 7% | 4.8% | 1.5% | 0.08% | 0.02% | | |
| Jun | 59% | 12% | 44% | 5% | 4.0% | 1.2% | 0.08% | 0.01% | | |
| Jul | 62% | 17% | 38% | 8% | 6.2% | 1.8% | 0.05% | 0.02% | | |
| Aug | 53% | 15% | 47% | 12% | 3.6% | 1.2% | 0.05% | 0.01% | | |
| Sep | 51% | 14% | 44% | 10% | 4.0% | 1.2% | 0.06% | 0.01% | | |
| Oct | 55% | 13% | 56% | 9% | 2.8% | 1.1% | 0.12% | 0.02% | | |
| Nov 16 | 74% | 18% | 54% | 11% | 7.0% | 1.8% | 0.09% | 0.02% | | |

Units: percent of total measured **organic carbon** (% OC)

**Table S3.** CMB Source Contribution of Fine Particulate Matter estimates of Organic Carbon (OC) and ions (Sulfate, Nitrate and Ammonium) by concentration (μg OC/m3) in Addis Ababa, November 2015 through November 2016.

| Month | PM mass | massunc | Sulfate | Sulfate std err | Nitrate | Nitrate std err | Ammonium | Ammonium std err | Dust | Dust unc | Biomass burning | Bb unc | Vehicles | vehunc | Residential coal combustion | coalunc | Fuel oil | fuel oil unc | Other PM | otherunc |
|---|---|---|---|---|---|---|---|---|---|---|---|---|---|---|---|---|---|---|---|---|
| Nov-15 | 39.92 | 7.71 | 2.59 | 0.13 | 0.32 | 0.04 | 0.64 | 0.10 | 5.02 | 1.23 | 5.47 | 1.11 | 8.52 | 1.15 | 1.86 | 0.43 | 0.26 | 0.06 | *15.24* | *7.98* |
| Dec-15 | 40.58 | 16.56 | 3.27 | 0.77 | 0.38 | 0.09 | 0.69 | 0.08 | 8.79 | 2.82 | 6.45 | 1.32 | 8.92 | 2.04 | 1.62 | 0.48 | 0.30 | 0.07 | *10.15* | *17.00* |
| Jan-16 | 45.37 | 37.39 | 3.24 | 0.68 | 0.36 | 0.01 | 0.91 | 0.13 | 6.87 | 3.14 | 8.01 | 1.63 | 8.29 | 1.61 | 2.06 | 0.59 | 0.21 | 0.03 | *15.43* | *37.61* |
| Feb-16 | 41.23 | 7.98 | 3.63 | 0.86 | 0.32 | 0.01 | 0.76 | 0.17 | 11.01 | 1.45 | 4.99 | 1.02 | 11.01 | 3.14 | 1.82 | 0.44 | 0.43 | 0.06 | *7.25* | *8.82* |
| Mar-16 | 40.89 | 9.13 | 2.23 | 0.24 | 0.19 | 0.07 | 0.70 | 0.27 | 11.39 | 1.42 | 5.53 | 1.12 | 9.53 | 1.67 | 1.74 | 0.41 | 0.29 | 0.07 | *9.28* | *9.47* |
| Apr-16 | 33.64 | 11.14 | 1.34 | 0.33 | 0.34 | 0.04 | 0.58 | 0.08 | 4.25 | 0.83 | 5.34 | 1.08 | 8.21 | 1.40 | 1.53 | 0.39 | 0.23 | 0.06 | *11.82* | *11.32* |
| May-16 | 35.35 | 12.99 | 1.74 | 0.35 | 0.39 | 0.04 | 0.46 | 0.15 | 13.24 | 5.08 | 6.46 | 1.31 | 11.17 | 1.92 | 1.22 | 0.38 | 0.24 | 0.05 | *0.43* | *14.15* |
| Jun-16 | 83.74 | 6.83 | 3.99 | 0.70 | 0.20 | 0.05 | 1.18 | 0.09 | 16.75 | 2.39 | 17.02 | 3.43 | 24.07 | 2.70 | 2.24 | 0.65 | 0.53 | 0.06 | 17.77 | 8.50 |
| Jul-16 | 70.13 | 17.80 | 2.89 | 0.41 | 0.26 | 0.09 | 1.25 | 0.22 | 6.58 | 1.32 | 19.32 | 3.88 | 22.03 | 2.70 | 3.72 | 0.78 | 0.35 | 0.09 | *13.73* | *18.49* |
| Aug-16 | 74.86 | 18.57 | 2.74 | 0.67 | 0.15 | 0.03 | 1.11 | 0.24 | 3.83 | 1.23 | 12.12 | 2.43 | 20.55 | 3.81 | 1.58 | 0.41 | 0.23 | 0.06 | *32.56* | *19.17* |
| Sep-16 | 61.11 | 13.01 | 4.61 | 0.41 | 0.20 | 0.06 | 2.32 | 0.14 | 5.88 | 1.22 | 13.55 | 2.72 | 22.48 | 3.36 | 2.06 | 0.46 | 0.34 | 0.06 | *9.67* | *13.77* |
| Oct-16 | 36.71 | 8.56 | 4.12 | 0.46 | 0.07 | 0.02 | 1.55 | 0.23 | 6.07 | 1.09 | 6.68 | 1.35 | 12.86 | 1.81 | 0.66 | 0.25 | 0.31 | 0.06 | *4.38* | *8.94* |
| Nov-16 | 39.49 | 7.56 | 3.38 | 0.34 | 0.05 | 0.03 | 0.69 | 0.17 | 4.33 | 0.42 | 10.49 | 2.11 | 14.61 | 2.59 | 1.93 | 0.43 | 0.27 | 0.04 | *3.74* | *8.29* |

Units: micrograms of **fine particulate matter** per cubic meter (μg $PM_{2.5}/m^3$)

*Blue italic text indicates* ***non-significant*** *values (criteria: concentration is* ***less than*** *2*uncertainty)*

**Table S4.** CMB Source Contribution of Fine Particulate Matter estimates of Organic Carbon (OC) and ions (Sulfate, Nitrate and Ammonium) by percent (%) in Addis Ababa, November 2015 through November 2016.

| Month | Sulfate | Sulfate std err | Nitrate | Nitrate std err | Ammonium | Ammonium std err | Dust | Dust unc | Biomass burning | bbunc | Vehicles | vehunc | Residential coal combustion | Coal unc | Fuel oil | fuel oil unc | Other PM | otherunc |
|---|---|---|---|---|---|---|---|---|---|---|---|---|---|---|---|---|---|---|
| Nov 15 | 6.5% | 1.3% | 0.8% | 0.2% | 1.6% | 0.4% | 13% | 4% | 14% | 4% | 21% | 5% | 4.7% | 1.4% | 0.6% | 0.2% | *38%* | *21%* |
| Dec | 8.0% | 3.8% | 0.9% | 0.4% | 1.7% | 0.7% | 22% | 11% | 16% | 7% | 22% | 10% | 4.0% | 2.0% | 0.7% | 0.4% | *25%* | *43%* |
| Jan | 7.1% | 6.1% | 0.8% | 0.6% | 2.0% | 1.7% | 15% | 14% | 18% | 15% | 18% | 15% | 4.5% | 4.0% | 0.5% | 0.4% | *34%* | *87%* |
| Feb | 8.8% | 2.7% | 0.8% | 0.2% | 1.8% | 0.6% | 27% | 6% | 12% | 3% | 27% | 9% | 4.4% | 1.4% | 1.0% | 0.3% | *18%* | *22%* |
| Mar | 5.5% | 1.4% | 0.5% | 0.2% | 1.7% | 0.8% | 28% | 7% | 14% | 4% | 23% | 7% | 4.3% | 1.4% | 0.7% | 0.2% | *23%* | *24%* |
| Apr | 4.0% | 1.6% | 1.0% | 0.4% | 1.7% | 0.6% | 13% | 5% | 16% | 6% | 24% | 9% | 4.5% | 1.9% | 0.7% | 0.3% | *35%* | *36%* |
| May | 4.9% | 2.1% | 1.1% | 0.4% | 1.3% | 0.6% | 37% | 20% | 18% | 8% | 32% | 13% | 3.5% | 1.7% | 0.7% | 0.3% | *1%* | *40%* |
| Jun | 4.8% | 0.9% | 0.2% | 0.1% | 1.4% | 0.2% | 20% | 3% | 20% | 4% | 29% | 4% | 2.7% | 0.8% | 0.6% | 0.1% | 21% | 10% |
| Jul | 4.1% | 1.2% | 0.4% | 0.2% | 1.8% | 0.6% | 9% | 3% | 28% | 9% | 31% | 9% | 5.3% | 1.7% | 0.5% | 0.2% | *20%* | *27%* |
| Aug | 3.7% | 1.3% | 0.2% | 0.1% | 1.5% | 0.5% | 5% | 2% | 16% | 5% | 27% | 8% | 2.1% | 0.8% | 0.3% | 0.1% | *43%* | *28%* |
| Sep | 7.5% | 1.7% | 0.3% | 0.1% | 3.8% | 0.8% | 10% | 3% | 22% | 6% | 37% | 10% | 3.4% | 1.0% | 0.6% | 0.2% | *16%* | *23%* |
| Oct | 11.2% | 2.9% | 0.2% | 0.1% | 4.2% | 1.2% | 17% | 5% | 18% | 6% | 35% | 10% | 1.8% | 0.8% | 0.9% | 0.2% | *12%* | *25%* |
| Nov 16 | 8.6% | 1.8% | 0.1% | 0.1% | 1.8% | 0.5% | 11% | 2% | 27% | 7% | 37% | 10% | 4.9% | 1.4% | 0.7% | 0.2% | *9%* | *21%* |
| Avg | 6.5% | 2.2% | 0.6% | 0.2% | 2.0% | 0.7% | 17.4% | 6.6% | 18.3% | 6.6% | 28.0% | 9.1% | 3.8% | 1.6% | 0.7% | 0.2% | 22.7% | 31.2% |
| Min | 3.7% | 0.9% | 0.1% | 0.1% | 1.3% | 0.2% | 5.1% | 2.1% | 12.1% | 3.4% | 18.3% | 4.0% | 1.8% | 0.8% | 0.3% | 0.1% | 1.2% | 10.3% |
| Max | 11.2% | 6.1% | 1.1% | 0.6% | 4.2% | 1.7% | 37.5% | 19.9% | 27.5% | 15.0% | 37.0% | 15.5% | 5.3% | 4.0% | 1.0% | 0.4% | 43.5% | 87.5% |

Units: percent of total measured **particulate matter** (% $PM_{2.5}$)

*Blue italic text indicates* ***non-significant*** *values (criteria: concentration is* ***less than*** *2*uncertainty)*
